# Goat Milk Protein-Derived ACE Inhibitory Peptide SLPQ Exerts Hypertension Alleviation Effects Partially by Regulating the Inflammatory Stress of Endothelial Cells

**DOI:** 10.3390/foods13213392

**Published:** 2024-10-25

**Authors:** Shenghao Xing, Xiaotong Zhang, Tong Mu, Jianxin Cao, Ke Zhao, Bing Han, Xinyan Peng

**Affiliations:** 1 College of Life Science, Yantai University, Yantai 264005, China; 2 Institute of Food Science, Zhejiang Academy of Agricultural Sciences, Hangzhou 310021, China; 3 College of Food Engineering and Nutritional Science, Shaanxi Normal University, Xi’an 710062, China

**Keywords:** goat milk, ACE inhibition, SLPQ, structure, activity, transcriptome

## Abstract

Hypertension has always posed a severe threat to people’s health. Food-derived angiotensin-converting enzyme (ACE)-inhibitory peptides have the potential to both prevent and treat hypertension. In the current investigation, two ACE-inhibitory peptides (SLPQ and PYVRYL) from goat milk were studied for their endothelial effects using EA.hy926 cells. PYVRYL outperformed SLPQ, yet neither impacted cell survival below 200 μg/mL. Investigation of SLPQ’s impact on EA.hy926 cell expression revealed 114 differentially expressed genes, with 65 downregulated and 49 upregulated. The genes were enriched in cytokine interactions, coagulation cascades, Hippo signaling, and ECM–receptor interaction. Decreased c-x-c motif chemokine ligand 2 (CXCL2), integrin subunit beta 2 (ITGB2), and fbj murine osteosarcoma viral oncogene homologue (FOS) expression and increased secreted phosphoprotein 1 (SPP1) expression may protect endothelial cells from inflammation. Our findings suggest that beyond ACE inhibition, SLPQ aids blood pressure control by influencing endothelial function, paving the way for its use as an antihypertensive food ingredient.

## 1. Introduction

Currently, hypertension is a chronic health problem affecting approximately 1 billion people worldwide [1]. It can put patients’ lives in jeopardy and raise their risk of stroke, heart disease, atherosclerosis, and other illnesses [2,3]. Angiotensin I-converting enzyme (ACE), sometimes referred to as kinase II or peptidyl carboxypeptidase, is an enzyme that carries a Zn^2+^ co-factor and is essential for controlling blood pressure in humans [4]. Angiotensin-converting enzyme (ACE) is a vascular endothelial cell membrane-bound enzyme that may release dipeptides from the C-terminus of various peptide substrates. Angiotensin I (Ang I) is converted to angiotensin II (Ang II) by ACE activity, which releases the His-Leu dipeptide located at its C-terminus [5]. Inhibiting the activation of the renin-angiotensin and bradykinin systems is the main goal of conventional pharmacological therapy for hypertension [6]. However, the risks associated with chemical medications in the treatment of high blood pressure cannot be ignored [7]. Natural products’ therapeutic usefulness has received increased attention due to their relative safety compared to chemical drugs [8]. Food-derived ACE-inhibiting peptides have attracted many researchers’ interest [9]. Fish, eggs, algae, dairy products, shellfish, and other natural sources have been used to explore angiotensin-converting enzyme (ACE)-inhibitory peptides [10,11,12].

Goat milk is a valuable source of nutrients, includes few allergens [13], and is easier to digest and absorb [14]. In addition, goat milk exerts antioxidant, anti-inflammatory, antiatherogenic, anticancer, and hepatoprotective properties [15,16,17]. The goat milk protein primarily consists of casein and whey protein, which contain several bioactive peptides [18,19]. Peptide formation can be attained in vitro through enzyme separation or in vivo via digestive enzymes like pepsin, trypsin, and intestinal microbial enzymes [20]. Certain bioactive peptides have two or more distinct biological activities and are multifunctional [21,22]. Due to their greater selectivity and safety compared to small-molecule drugs, peptides are currently being investigated for their potential medical applications. Indeed, over 60 peptides have been approved and marketed as drugs by the Food and Drug Administration as of now [23]. However, the studies on the potential application of antihypertension peptides derived from goat milk are still unclear, and more research is required to explore their toxicity and biological function.

SLPQ and PYVRYL are two previously reported ACE-inhibitory peptides derived from goat β-casein [24,25]. Ibrahim et al. reported that goat casein and whey protein contain novel, potent ACE-inhibiting peptides that can be released by gastric pepsin [26,27]. Those peptides exert a wide range of physiological activities, e.g., antioxidant [28], antitumor, and anti-inflammatory, and regulate the balance of gut microbiota [29].

ACE-inhibitory peptides have been extensively investigated, and there are a lot of studies that reported ACE-inhibitory peptides originating from goat milk [30]. In particular, Parmar et al. used *Lactobacillus fermentum* (LF) and *Lactobacillus casei* (NK9) to ferment goat milk, and 26 peptides with ACE-inhibitory activity were identified and characterized [31]. In addition, 16 ACE-inhibitory peptides were identified from caprine kefir, and among them, PYVRYL showed the highest ACE-inhibitory activity [24]. SLPQ is another ACE-inhibitory peptide reported previously, which was isolated through a rigorous purification process from the hydrolysate of goat milk protein [25]. Given its inhibitory action against ACE and possible role in the hydrolysate’s overall bioactivity, it is worth further investigating its effects on blood pressure management and cardiovascular health. The endothelium, a monolayer of endothelial cells (ECs), forms the vital interface between blood and tissue and regulates vascular function along the luminal surface of blood vessels in humans [32]. The growth factors and signals originating from the microenvironment of tissue can affect the adaptations of endothelial cells (ECs) [33]. Therefore, ECs carry out multiple biological and physiological functions in tissue, such as the regulation of vascular tone, platelet aggregation, angiogenesis, leukocyte trafficking, and other responses to inflammation. ECs produce a variety of vasoregulatory and pro-angiogenic molecules, and EA.hy926 is a useful model for studying angiotensin-converting enzyme (ACE) biology [34,35]. EA.hy926 cells were selected for additional examination to ascertain the regulatory effects of ACE-inhibitory peptides on endothelial cells. Exogenous intervention has extremely complex impacts on cell biology, but under certain conditions, the RNA sequencing (RNA-seq) approach offers a compelling and useful means of elucidating the biological response at the transcriptional level [36]. Considering the relatively high ACE-inhibitory activity and distinct structure of the two peptides mentioned above (PYVRYL and SLPQ), their potential role in the function of endothelial cells attracted our attention.

Therefore, the current study aimed to investigate the effects and regulatory mechanism of the goat milk protein-derived ACE-inhibitory peptides SLPQ and PYVRYL on endothelial cells. The inhibitory activity and toxicity of SLPQ and PYVRYL were first evaluated, and then a possible blood pressure-lowering effect was determined by tracking nitric oxide (NO) production and endothelial nitric oxide synthase (eNOS) expression. Furthermore, transcriptomic profiling of cells treated with SLPQ or not was analyzed to clarify its potential antihypertension mechanism. The results will provide a theoretical basis for the application of goat milk-derived peptides in functional food production.

## 2. Materials and Methods

### 2.1. Materials and Reagents

SLPQ and PYVRYL were generated by Shanghai QiangYao Biology, with a purity higher than 95%. EA.hy926 cells were acquired from the Chinese Academy of Sciences stem cell bank (Shanghai Academy of Biological Sciences, Shanghai, China). ThermoFisher Scientific (Waltham, MA, USA) provided Dulbecco’s modified Eagle’s medium (DMEM). Invitrogen Co. provided fetal bovine serum (FBS) and streptomycin–penicillin (Carlsbad, CA, USA). Thiazolyl blue tetrazolium bromide (MTT) was purchased from Biorigin (Beijing, China). Sigma-Aldrich Chemical Co. provided N-hippuryl-His-Leu hydrate (HHL) and ACE (St. Louis, MO, USA). The remaining reagents were analytical or HPLC grade.

### 2.2. Assay of ACE-Inhibitory Activity

ACE-inhibitory activity was assayed following a previous study with slight modification [37]. Briefly, SLPQ, PYVRYL, and HHL powders were first dissolved in 100 mM borate buffer (pH 8.3) containing 300 mM NaCl. ACE (2 mU) was placed at 37 °C for 5 min to allow for full reaction. A mixture of 10 μL peptide solution and 50 μL HHL substrate (5 mM) was incubated at 37 °C for 5 min. ACE (2 mU) was then added and incubated at 37 °C for another 30 min. A culture temperature 37 °C is standard for mammalian cells (including EA.hy926 cells). At this temperature, cells are able to maintain normal metabolic and physiological functions. Therefore, pre-incubating them at 37 °C may help them to better bind or function with the cells. To halt the reaction, 100 μL of 1 M HCl was added. Following a 20 seconds’ vortexing with 1 mL of ethyl acetate, hippuric acid was extracted. It was then centrifuged at 8000× *g* rpm for 15 min and left to stand for 5 min. After gathering 800 μL of the ethyl acetate layer, the samples were dried in a drying oven. The solution of samples was then sonicated to promote solubilization, and its absorbance at 228 nm was measured. ACE inhibition was calculated using the formula below:

ACE inhibition (%) = [(B − A)/(B − C)]

where A represents the absorbance value measured in the experimental group, B represents the absorbance value measured in the control group, and C represents the absorbance value measured in the blank group.

### 2.3. Molecular Docking Analysis and Three-Dimensional (3D) Structure Prediction

The 3D structural models of the identified peptides were first generated using the PEP-FOLD tool V3.5 (http://bioserv.rpbs.univ-paris-diderot.fr/services/PEP-FOLD3, accessed on 20 November 2021), which predicts the 3D structural model of the identified peptides based on the available amino acid sequences by selecting the folding mode with the lowest energy [38]. Molecular docking analysis of SLPQ and PYVRYL was performed using the semi-flexible program CDOCKER in Discovery Studio 2019 software (Accelrys Software Inc., San Diego, CA, USA). The crystal structure of the human ACE complex (PDB:1O8a) was downloaded from the PDB database. The Prepare Program was applied to remove water molecules as well as ligands and then undertake pre-processing, such as cycling and protonation. Possible active binding sites were defined in the receptor cavity, while the three-dimensional structure of the predicted peptide ligand was hydrogenated and minimally energized. Molecular docking results were evaluated based on the -CDOCKER interaction energy (-CIE) score, interaction site, and type of interaction, as depicted in our previous study [39,40].

### 2.4. Cell Culture and MTT Analysis

The cells were cultured in DMEM with 100 units/mL streptomycin–penicillin plus 2% FBS under an atmosphere of 5% CO_2_, 37 °C, and 100% humidity. The cells were seeded in 96-well plates at a density of 1 × 10^5^ cells/well, and then treated with different concentrations of SLPQ (0, 12.5, 25, 50, 100, 200, 400, 800 μg/mL) or PYVRYL (0, 25, 50, 100, 200, 400, 800 μg/mL) for 12 or 24 h. After that, 0.05 mg/mL of MTT was added to each well and the plate was incubated for four hours at 37 °C. Finally, the culture media were replaced with 150 μL DMSO, and the OD value was detected at a wavelength of 570 nm (Infinity F200 microplate reader, Mainz, Germany).

### 2.5. Determination of NO

Extracellular NO production was measured using an NO assay kit (Beyotime, Wuhan, China). Briefly, EA.hy926 cells were inoculated in 96-well plates (1 × 104 cells/well) for 48 h and then starved for 24 h in culture medium without FBS. After that, the cells were treated with different concentrations (0, 50, 100, and 200 μg/mL) of SLPQ or PYVRYL for 12 h and 24 h. Then, 100 μL of culture medium was mixed with 50 μL of Grice’s reagent I and 50 μL of Grice’s reagent II and then incubated for 10 min at room temperature. Absorbance was measured at 540 nm, and fresh culture medium was used as a blank for each experiment. The total protein content of the cells in each well was determined using a BCA kit (Thermo Fisher Scientific, Waltham, MA, USA) and applied to correct the NO content.

### 2.6. Western Blotting

Cells treated with SLPQ and PYVRYL peptides for 12 h were harvested for assessment of protein expression. Total protein was extracted using the protein extraction kit and protein concentration was determined using the BCA kit (Thermo Fisher Scientific, Waltham, MA, USA). Proteins were separated on 12.5% SDS-PAGE gel and transferred to a polyvinylidene fluoride (PVDF) membrane using a Bio-Rad wet transfer device (Hercules, CA, USA). At the end of the transfer, the PVDF membranes were put in block solution at 4 °C for 2 h. Subsequently, the membranes were incubated overnight at 4 °C with primary antibodies of human endothelial nitric oxide synthase (eNOS) (Proteintech Co., Ltd., Shenzhen, China). After washing with Tris-buffered saline with Tween (TBST) buffer 3 times (10 min/time), the membrane was incubated with the secondary antibody for 2 h at room temperature and washed 3 times with TBST (10 min each). Finally, an ECL solution (Tanon Co., Ltd., Shanghai, China) was configured according to the kit instructions, and the membranes with drops of the luminescent solution were placed into a chemiluminescence instrument for development. Images were captured using chemiluminescence (Tanon Co., Ltd., Shanghai, China) and grayscale scanning analysis was conducted.

### 2.7. RNA-Seq and Data Analysis

Total RNA was extracted using a Trizol reagent following the manufacturer’s instructions. Next, genomic DNA was eliminated using DNaseI (Takara Bio Inc., Kusatsu, Japan), and the quality and integrity of extracted RNA were assessed using an Agilent 2100 (Agilent Technologies, Palo Alto, CA, USA) and NanoDrop ND-2000 (Thermo Scientific, Waltham, MA, USA). RNA libraries were generated in compliance with the guidelines provided by Illumina, San Diego, CA, USA’s TruSeqTMRNA sample preparation kit. Following PCR enrichment, the cDNAs were then sequenced using IlluminaHiSeq X10 (paired-end library, read length 2 × 150 bp) and quantified using TBS380 (Picogreen, Turner BioSystems, Bay Area, CA, USA). Using RNA-seq by expectation maximization (RSEM) software (version 1.3.1), the expression levels of genes and transcripts were measured following a quality screening of the raw sequencing data. DESeq2 was used to analyze the differentially expressed (DE) genes with a cutoff of absolute fold change (FC) > 1.5 and false-discovery rate (FDR) < 0.05. Gene Ontology (GO) and Kyoto Encyclopedia of Genes and Genomes (KEGG) enrichment were then performed utilizing differential gene function enrichment analysis. Multiple tests were performed using the BH (FDR) method, and significant enrichment was considered to exist when *p* ≤ 0.15 for gene ontology (GO) and *p* ≤ 0.1 for Kyoto Encyclopedia of Genes and Genomes (KEGG) were met.

### 2.8. Statistical Analysis

The data analysis was conducted using GraphPad Prism (version 9.0), Origin (version 9.0), and SPSS Statistics 19.0 (version 19.0). The results are displayed as means ± standard deviation (SD). One-way analysis of variance (ANOVA) was used to analyze the data on ACE inhibition, cell viability, and relative gene expression. Dunnett’s multiple comparisons posttest was then used to assess the results. The sequencing counts per million (CPM) and the Western blot data were analyzed using the *t*-test. *p* < 0.05 represents a significant difference. Three biological replicates were conducted in each experiment, and each experiment was repeated 3 times.

## 3. Results

### 3.1. ACE-Inhibitory Activity and Interactive Mechanism of SLPQ and PYVRYL Against ACE

**Figure 1:**
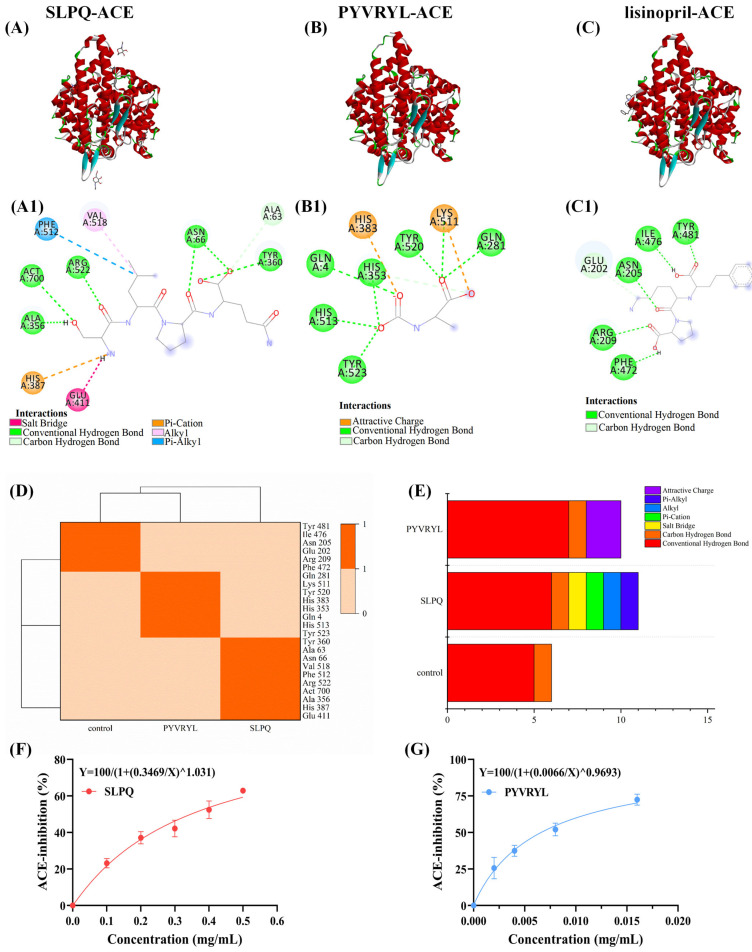
(**A**–**C**) Molecular docking model of interaction between ACE-inhibitory peptide and ACE (PDB ID: 1O86). Structure of SLPQ (A1), PYVRYL (B1), and Lisinopril (C1) in complex with ACE protein 1O8a 2D structure. (**D**) Number of amino acid residues in the docking site with the ACE-inhibitory peptide. (**E**) Statistics of different chemical bonds formed after docking with ACE-inhibitory peptide. (**F**,**G**) Results of ACE-inhibitory activity of milk-derived SLPQ, PYVRYL peptides.

The molecular interactions and binding affinity between ACE and peptide-binding sites have been clarified through molecular docking studies [41]. Figure 1A–C display the 3D and 2D structures of SLPQ, PYVRYL, and lisinopril with ACE (PDB: 1O86). There are a total of six conventional hydrogen bonds (Ala 356, Act 700, Arg 522, Asn 66, and Tyr 360) and two salt bridges (His 387 and Glu 411) between SLPQ and ACE. During PYVRYL’s interaction with ACE, seven hydrogen bonds were seen (Tyr 523, His 513, Gln 4, His 353, Tyr 520, Lys 511, and Gln 281). A total of five hydrogen bonds (Phe 472, Arg 209, Asn 205, Ile 476, and Tyr 481) stabilized the lisinopril–ACE complex (Figure 1(A1–C1)). Figure 1D,E show the number of amino acid residues on the docking site with ACE-inhibitory peptide and the statistics of different chemical bonds formed after docking with ACE-inhibitory peptide. According to the majority of research, hydrogen bonds are crucial for the binding of inhibitors to ACE [42]. Therefore, PYVRYL exhibits higher activity than SLPQ when it comes to exerting its inhibitory effect on the active site of ACE. SLPQ and PYVRYL have minimum binding energies of −7.2 kcal/mol and −13.5 kcal/mol, respectively, with ACE. Low binding energy indicates stable ACE ligand binding [43], implying that SLPQ and PYVRYL can bind tightly to ACE and perform ACE-inhibition functions.

As shown in Figure 1F,G, both SLPQ and PYVRYL peptides exhibited ACE-inhibitory activity. Specifically, the IC50 value of ACE inhibition was 347 μg/mL and 7 μg/mL for SLPQ and PYVRYL, respectively. The result was similar to a previous study that identified SLPQ from goat milk hydrolysate (IC50 values of 316–354 μmol/L) [25]. Quiro et al. reported ACE-inhibitory properties of kefir made from caprine milk, with PYVRYL showing potent ACE-inhibitory activity with IC50 of 2.4 μM, which was much higher than that of SLPQ [24].

### 3.2. Cellular Toxicity and Effects of SLPQ and PYVRYL on NO Synthesis

**Figure 2:**
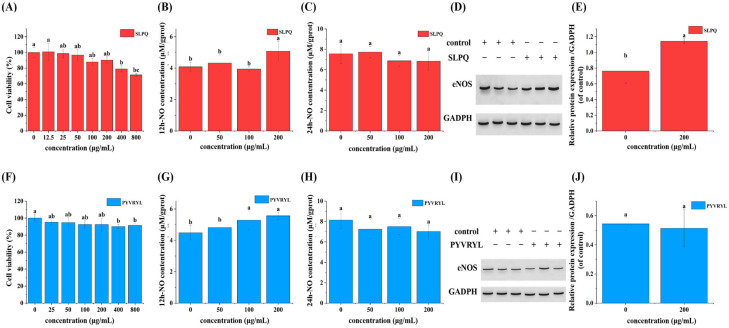
(**A**,**F**) Effects of SLPQ and PYVRYL on EA.hy926 cell viability. (**B**,**C**,**G**,**H**) Effects of SLPQ and PYVRYL on NO production in EA.hy926 cells. (**D**,**I**) Immunoblotting results of eNOS protein expression in EA.hy926 cells with ACE-inhibitory peptide intervention. (**E**,**J**) Densitometry of eNOS protein expression in EA.hy926 cells with ACE-inhibitory peptide intervention. Data are presented as means ± SD. Different letters indicate significant differences (*p* < 0.05).

The viability of EA.hy926 cells was not affected by supplementation of SLPQ or PYVRYL with a concentration lower than 200 μg/mL, while higher doses of the two peptides (400 and 800 μg/mL) both showed obvious toxic effects on the cells (Figure 2A,F). Consequently, peptides SLPQ or PYVRYL were supplemented at concentrations ranging from 0 to 200 μg/mL in the following study. The bradykinin–NO signaling pathway can be activated by angiotensin II [44], and NO plays an important role in regulating blood pressure and the tension of vascular smooth muscle [45]. Therefore, the content of NO in the cells treated with different concentrations of SLPQ (0, 50, 100, and 200 μg/mL) or PYVRYL (0, 50, 100, and 200 μg/mL) was detected. As shown in Figure 2B,C,G,H, compared with the control group, supplementation of SLPQ (200 μg/mL) or PYVRYL (100 and 200 μg/mL) significantly elevated the NO content (*p* < 0.05). NO is generated in the endothelial cells and plays an important role in vasodilatation and blood pressure control. Vasorelaxation is largely classified into endothelium-dependent and -independent vasorelaxation [46]. Endothelial nitric oxide (eNOS)-mediated nitric oxide (NO) production in the endothelium is one of the major mechanisms of vasorelaxation [47], and is capable of an endothelium-dependent vasorelaxant effect in hypertension [48]. Most recently, the potential vasodilator function of another goat milk-derived ACE-inhibitory peptide, SQPK, was reported, which could elevate NO production and eNOS expression [39]. However, dysregulation of NO can lead to uncontrolled inflammation and tissue damage [49]. Interestingly, supplementation of 200 μg/mL SLPQ instead of PYVRYL dramatically elevated the protein expression of eNOS (Figure 2D,E,I,J). Considering the important role of eNOS in regulating NO synthesis and the stimulatory effects of SQPK on eNOS expression, RNA-seq was applied in the following study to further explore the regulatory mechanism of SLPQ on the function of endothelial cells.

### 3.3. Potential Antihypertensive Mechanism of SLPQ by RNA-Seq

#### 3.3.1. SLPQ-Regulated Transcriptomic Profiling of Endothelial Cells

**Figure 3:**
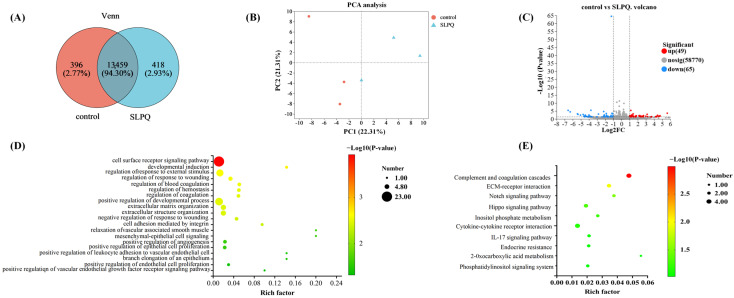
(**A**) Venn diagram of expressed genes in the control and SLPQ groups. (**B**) Principal component analysis of expressed genes under the two treatments. (**C**) Volcano plot of differentially expressed genes: downregulated genes are marked in green, while upregulated genes are marked in red. (**D**) GO enrichment analysis. (**E**) KEGG enrichment analysis results.

RNA-seq was applied to evaluate the genome-wide gene expression profiling of endothelial cells treated with SLPQ or not for 12 h (200 μg/mL). The transcriptome results showed that the GC contents ranged from 50.17 ± 0.02% to 50.77 ± 0.02%, and the number of raw reads ranged from 49.86 million to 67.57 million (Table S1). As shown in Figure 3A, after being mapped to the human genome (GRCh38), 13,855 and 13,877 genes were observed in the control and SLPQ group, respectively. Among them, most genes (*n* = 13,293) were commonly expressed in the two groups. Principal component analysis (PCA) results showed that the transcriptomic profiling of SLPQ and control groups showed clear clustering, suggesting that the intervention of SLPQ did affect the genome profiling of the cells. In addition, the DESeq2 was used for the selection of differentially expressed (DE) genes (FDR < 0.05 and |fold change| > 1.5). The results showed that 114 DE genes were obtained between the control and SLPQ groups, with 49 genes being upregulated and 65 downregulated (Figure 3C).

To understand the role of these DE genes, GO (Gene Ontology) and KEGG (Kyoto Encyclopedia of Genes and Genomes) pathway analyses were applied for functional enrichment. Among the significantly enriched 20 GO terms (*p* < 0.05), 14 GOs are closely related to vascular functions (Figure 3D). Interestingly, 8 out of the 14 GOs were targeted endothelial functions, such as “relaxation of vascular associated smooth muscle”, “mesenchymal-epithelial cell signaling”, “positive regulation of angiogenesis”, “positive regulation of epithelial cell proliferation”, “positive regulation of leukocyte adhesion to vascular endothelial cell”, “branch elongation of an epithelium”, “positive regulation of endothelial cell proliferation”, and “positive regulation of vascular endothelial growth factor receptor signaling pathway”. Considering the important role of blood vessels and endothelial cells in vasodilatation and blood pressure control [46,47,48], those GO terms were further analyzed.

In addition, the KEGG pathway results showed that 7 out of the 10 enriched pathways were related to an inflammatory response (*p* < 0.1) (Figure 3E), including “Complement and coagulation cascades”, “ECM-receptor interaction”, “Notch signaling pathway”, “Hippo signaling pathway”, “Cytokine–cytokine receptor interaction”, “Interleukin 17A (IL-17A) signaling pathway”, and “Endocrine resistance”. Recent studies have demonstrated the strong correlations between endothelial dysfunction and the inflammatory process [50,51]. A connection between the Hippo pathway and other key signaling pathways involved in immune regulation has also been confirmed, including transforming growth factor β (TGF-β) and Toll-like receptor (TLR) signaling pathways. Research has revealed that interleukin 17A (IL-17A) effectively triggers the activation of mitogen-activated protein kinase (MAPK) and downstream transcription factors activating protein activator protein 1 ((AP)-1) and p65 nuclear factor kappa-light-chain-enhancer of activated B cells (NF-κB), thereby intensifying and sustaining the inflammatory response [52]. Therefore, those seven inflammation-related pathways were selected for further analysis, aiming to clarify the potential antihypertensive mechanism of SLPQ.

#### 3.3.2. Gene Network Analysis

**Figure 4:**
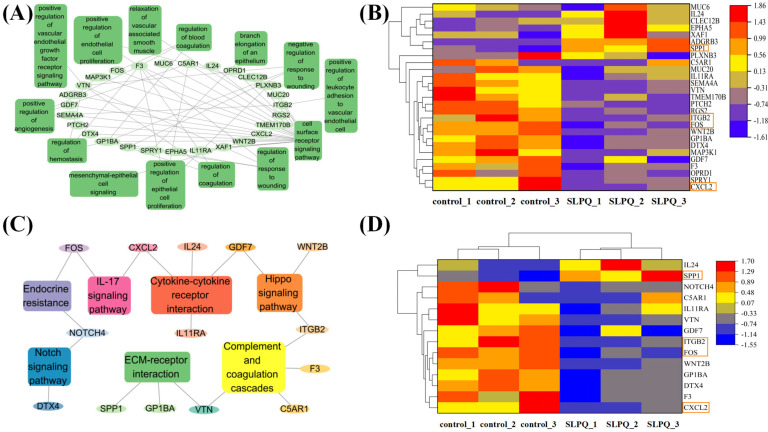
(**A**) Interaction network of the GO enrichment analysis involved in the related pathways. (**B**) GO heatmap of 14 genes’ targeted endothelial functions. Orange boxes: highlight. (**C**) Interaction network of the DE genes involved in the top 7 inflammation-related pathways. (**D**) Heatmap of expressed DE genes involved in the top 7 inflammation-related pathways (FDR < 0.05, and absolute FC > 2). Orange boxes: highlight.

As shown in Figure 3E, the DE gene interaction network is involved in the seven immune related KEGG pathways. Figure 4C,D illustrates the inflammatory metabolism-related differentially expressed genes in the KEGG enrichment results. Interestingly, the gene network analysis for GO showed similar results to that for KEGG (Figure 4A,B). In comparison to the control group, it was found that after SLPQ treatment, the expression of the c-x-c motif chemokine ligand 2 (CXCL2), integrin beta 2 (ITGB2), and fos proto-oncogene (FOS) genes was decreased and the expression of the secreted phosphoprotein 1 (SPP1) gene was increased compared with the control group. Macrophage inflammatory protein (MIP)-2, or CXCL2, is a member of the CXC chemokine family and binds specifically to c-x-c chemokine receptor type 2 (CXCR2). Bioinformatic analysis has demonstrated in recent research that CXCL2 and PAA formation are closely associated [53]. By inhibiting CXCL2–CXCR2, inflammation may be reduced through the AKT and signal transducer and activator of transcription 3 (STAT3) pathways. Osteopontin (OPN), or SPP1, is a coding protein that is regarded to be a crucial cytokine that aids in the expansion of immune cells and type 1 cytokine expression at inflammatory sites [54,55]. SPP1, for instance, has a role in both acute and chronic neuritis [56] and may promote pulmonary neutrophil accumulation to mediate transfusion-related acute lung injury (ALI) [57]. Wu et al. additionally verified that ALI had elevated SPP1 expression and that boosting SPP1 could prevent XBJ’s protective effect on ALI.

**Figure 5:**
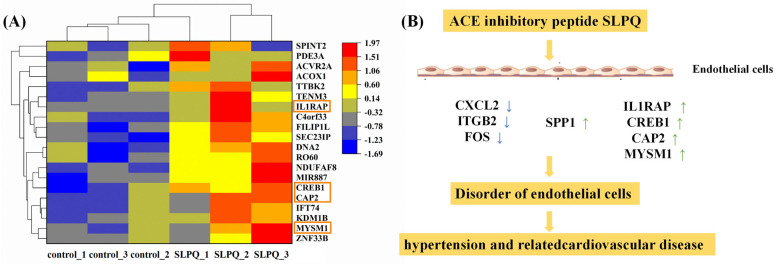
(**A**) Heatmap of the 20 hypertension-related genes most affected by SLPQ treatment. Orange boxes: highlight. (**B**) Network of DE genes contributing to endothelial cell function. Arrow: process of up– or down–regulation of gene expression.

Figure 5A displays the 20 hypertension-related DE genes that were most affected by SLPQ intervention. The 11,822 hypertension-related genes were downloaded from Pathcards (https://www.genecards.org/, accessed on 1 April 2024). Among them, some genes are also closely related to the immune function. Specifically, interleukin 1 receptor accessory protein (ILIRAP) was specifically expressed in stromal cells, where IL1 beta also promoted the expression of inflammatory chemokines and cytokines [58]. Myb-like swi/snf complex member 1 (MYSM1) is a key suppressor of innate immunity and autoimmunity and represents a potential therapeutic agent for infectious, inflammatory, and autoimmune diseases [59]. CAMP-responsive element-binding protein 1 (Creb1) is a transcription factor that can be induced by a variety of growth factors and inflammatory signals to regulate the expression of genes containing a cAMP-responsive element such as Il-6 [60,61]. Adenylate cyclase-associated protein 2 (CAP2), a regulator of the actin cytoskeleton, has roles in cell migration and wound healing [62].

Overall, SLPQ partially alleviated endothelial cell dysfunction through the inflammation-related signaling pathways, and some genes, such as CXCL2, ITGB2, FOS, SPP1, ILIRAP, MYSM1, and Creb1 might play important roles in the process (Figure 5B). However, the fact that not all the sequencing results were validated is an obvious limitation of the current study, and validation and cross-talk of those pathways and genes will be conducted in our future studies.

## 4. Conclusions

This study found that PYVRYL had higher inhibitory efficacy than SLPQ, while SLPQ significantly increased NO production and eNOS expression (*p* < 0.05). Furthermore, the research revealed that the DE genes triggered by SLPQ treatment were concentrated in specific pathways. Consequently, SLPQ holds the potential to mitigate endothelial cell dysfunction, at least partially, by regulating cytokine–cytokine receptor interactions, complementation and coagulation cascades, Hippo signaling pathways, and ECM–receptor interactions. Additionally, research reveals that the DE genes activated by SLPQ therapy were enriched to influence complementation and coagulation cascades, Hippo signaling pathways, cytokine–cytokine receptor interactions, and ECM–receptor interactions. SLPQ may be able to partially alleviate endothelial cell dysfunction. Additionally, the increased expression of SPP1 and the decreased expression of ITGB2, FOS, and CXCL2 genes may help shield endothelial cells from inflammatory stress. Overall, our findings demonstrate that aside from the ACE-inhibitory effect, the goat milk-derived peptide SLPQ could help with blood pressure control by influencing endothelial function. This study lays the groundwork for the use of the SLPQ peptide as a functional food ingredient with antihypertensive properties.

## Data Availability

The original contributions presented in the study are included in the article/Supplementary Material, further inquiries can be directed to the corresponding author.

## Supplementary Materials

The following supporting information can be downloaded at https://www.mdpi.com/article/10.3390/foods13213392/s1. Table S1: Statistics of sequencing data.
