# Supplementary material for: Goat Milk Protein-Derived ACE Inhibitory Peptide SLPQ Exerts Hypertension Alleviation Effects Partially by Regulating the Inflammatory Stress of Endothelial Cells
Source: Foods. 2024 Oct 25;13(21):3392. doi: 10.3390/foods13213392 (PMC11545510; doi:10.3390/foods13213392)
Supplement: Supplementary file 1 [file foods-13-03392-s001.zip › foods-3232956-supplementary.pdf]

Supplementary Material

Table S1. Statistics of sequencing data.

Sample	Raw reads	Clean reads	Error rate(%)	Q20(%)	Q30(%)	GC content(%)
control_1	67565966	67174452	0.0245	98.2	94.61	50.55
control_2	49856360	49543260	0.0248	98.1	94.33	50.45
control_3	54885834	54545828	0.0245	98.21	94.61	50.69
SLPQ_1	59034862	58684088	0.0245	98.2	94.61	50.57
SLPQ_2	58226316	57907994	0.0244	98.28	94.74	50.27
SLPQ_3	51299248	50929996	0.0246	98.16	94.54	50.43
